# From Africa to the World: Reimagining Africa’s research capacity and culture in the global knowledge economy

**DOI:** 10.7189/jogh.10.010321

**Published:** 2020-06

**Authors:** Isaac Iyinoluwa Olufadewa, Miracle Ayomikun Adesina, Toluwase Ayorinde

**Affiliations:** 1 Slum and Rural Health Initiative Research Academy, Ibadan, Nigeria; 2 University of Ibadan, Ibadan, Nigeria; 3 Pan African University of Life and Earth Sciences Institute, Ibadan, Nigeria

Africa sits on a keg of gun powder. Despite having over 15% of the world’s population and about a quarter of the global burden of disease, she has less than 3% of the world’s health care professionals and barely 1% of global research output [1]. This is not surprising as Sub-Saharan Africa averagely contributes about 0.4% of its Gross Domestic Product (GDP) to scientific research [2], while Europe, Asia, and North America contribute about 27%, 31% and 37% respectively to research and development. Majority of West African countries spend less than 0.25% of their GDP on Research and Development while most east and southern African countries spend 1% or less (**Table 1**) [1]. Furthermore, collaboration between sub-Saharan African countries is alarmingly low – the World Bank provided evidence that collaboration between researchers is between 0.9% in west and central Africa to 2.3% in southern Africa [4].

**Table 1:** Gross domestic expenditure on R&D (GERD) as a percentage of GDP, 2017 or latest available year for sub-Saharan Africa*

Country	Region	GERD
Nigeria	West Africa	0.22%
Ghana	West Africa	0.38%
Burkina Faso	West Africa	0.67%
Mali	West Africa	0.29%
Gabon	Central Africa	0.58%
Democratic Republic of the Congo	Central Africa	0.41%
Uganda	East Africa	0.17%
Kenya	East Africa	0.79%
Tanzania	East Africa	0.53%
Zambia	East Africa	0.29%
Mozambique	Southern Africa	0.34%
Namibia	Southern Africa	0.34%
Botswana	Southern Africa	0.54%
South Africa	Southern Africa	0.82%
Lesotho	Southern Africa	0.05%
Madagascar	East Africa	0.01%

GDP – gross domestic product

*Source: World Bank data [3].

Also, there is an acute shortage of adequately trained research leaders in the continent who can empower, mentor and create more opportunities for young African researchers to flourish [5]. Consequently, there is a huge gulf in research capacity and infrastructure which impedes Africa’s ability to effectively deal with the root causes of its deplorable state of health, unemployment, poverty and other indices of development. How can a continent rise above the level of its contribution, collaboration and competencies? Undoubtedly, these persistent problems have contributed to the weak health care systems in many African countries which is occasionally threatened by outbreaks such as Ebola, slow decline in tuberculosis, maternal and infant mortality which are largely preventable and stalled progress towards the attainment of the Sustainable Development Goals, SDGs in Africa.

Another perturbing issue is the practice of ‘helicopter science’ or ‘parachute study’ where foreign researchers harvest samples or data from Africa and analyse in their countries with little input from or credit given to local African researchers and communities who aid the work is still on-going. Some African researchers do not even contribute to the design or activities of the projects of international health researchers that take place in their ‘backyards.’ If African governments, research institutions, health care organizations, funding partners and friends of Africa keep following the ‘business-as-usual’ approach, we are following a blueprint for disaster.

Africa is a very unique continent with highly diverse societies, cultures, religions, traditions, and beliefs which are remarkably different from that of the western or developed world. Africa-based initiatives and organizations understand these factors, which are the root causes of many of the health and social challenges facing Africa and should be at the forefront of these studies. Researches on issues in Africa should be led by Africans for the advancement of the African continent. Foreign researches on Africa often become “spectacular failures” in the short-term or in the long run if they do not effectively engage or trust the leadership of African researchers. The Human Heredity and Health in Africa (H3Africa) is changing this sad narrative in the field of Genomics. H3Africa which is funded by the US National Institutes of Health and the London-based Wellcome Trust has sponsored several genomics studies whose principal investigators are African. Just last year, H3Africa published a guide for the ethical handling of genomic research and biobanking in Africa that empowers local researchers with the needed information to have more control over genomic studies and foreign research partners and sponsors with equitable rules of engagement for research in Africa [6]. More inclusive policies and guidelines like these from coordinating or funding bodies are vital in promoting research equity and shifting control of international studies conducted in Africa to African researchers.

**Figure:**
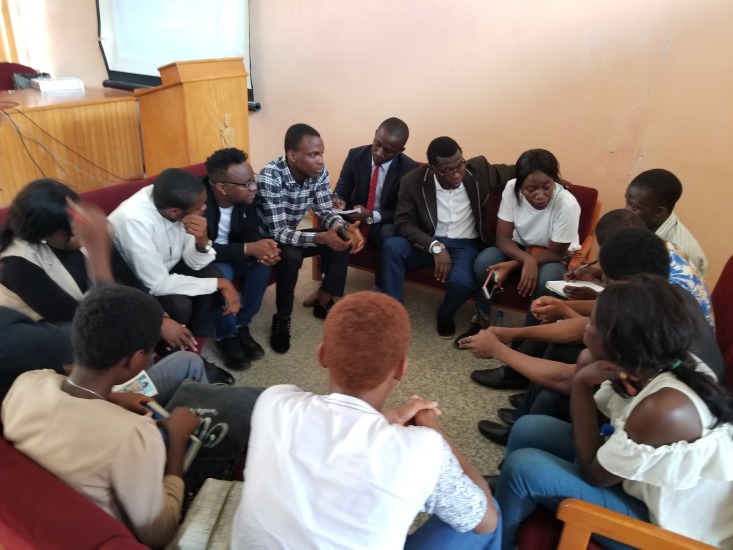
Photo: Young African researchers discussing during a capacity-building workshop at Slum and Rural Health Initiative's annual Young Einstein Summit (source: Slum and Rural Health Initiative, used with permission).

At the international level, there is the need for the United Nations and the African Union Commission to work with global research institutions and African-based research academies to promote equitable research policies and ensure adequate policy advocacy to ensure mutually beneficial global collaborations and advocate for better funding for research especially for research-intensive universities on the African continent.

At the regional level, inter-African research collaborations should be significantly improved which can be achieved through inter-African networking and fellowship opportunities. Also, as suggested in a meeting of The Consortium for Advanced Research Training in Africa (CARTA) Vice-Chancellor and heads of partner institutions, research-intensive universities have to be identified, strengthened and funded to train researchers, upgrade research facilities among other strategies. For the research climate to be more favourable for African researchers, the region needs to expel hierarchical, traditional and conventional models that paint a portrait of neo-colonial systems that are inequitable and unfair.

At the country and local level, there needs to be more investment in research – that will help to fund local research projects, infrastructure and capacity-building for local researchers. It is high time Africa looked inwards to locally-led and run research initiatives such as the African Doctoral Dissertation Research Fellowship (ADDRF) program and the Slum and Rural Health Initiative Research Academy. There is also a need to ensure inclusive laws and policies that will involve the participation of local ethical committees that will empower and prepare African researchers for international collaborative research projects that will now be mutually beneficial.

Brain Greenwood, a British researcher who worked in Africa for over four decades before winning the prestigious Gairdner Global Health Award in 2012 words are instructive, “…I have been convinced that research on the health problems of Africa should be led by African scientists” [7]. I hope Africa and the world do not only listen to Greenwood’s wise words but take action to avert the looming time-bomb.
